# Upstaging of screen-detected lung cancers during diagnostic assessment

**DOI:** 10.1136/thorax-2025-224006

**Published:** 2026-02-02

**Authors:** Monica L Mullin, Priyam Verghese, Chuen R Khaw, Andrew Creamer, Amyn Bhamani, Ruth Prendecki, Jennifer L Dickson, Carolyn Horst, Sophie Tisi, Helen Hall, Kylie Gyertson, Esther Arthur-Darkwa, Laura Farrelly, John McCabe, Ricky Thakrar, Arjun Nair, Anand Devaraj, Neal Navani, Allan Hackshaw, The SUMMIT consortium, Aashna Samson, Sam M Janes

**Affiliations:** 1 Lungs for Living Research Centre, UCL Respiratory, University College London, London, UK; 2 Respirology, The University of British Columbia, Vancouver, British Columbia, Canada; 3 Basildon University Hospital, Basildon, UK; 4 University College London Hospitals NHS Foundation Trust, London, UK; 5 Cancer Research UK & UCL Cancer Trials Centre, University College London, London, UK; 6 MRC Clinical Trials Unit at UCL, London, UK; 7 Radiology, University College Hospital, London, UK; 8 Radiology, Royal Brompton and Harefield NHS Foundation Trust, London, UK

**Keywords:** Lung Cancer, Non-Small Cell Lung Cancer, Referral and Consultation, Thoracic Surgery

## Abstract

**Introduction:**

Lung cancer is the leading cause of cancer-related death worldwide. Low-dose CT (LDCT) screening improves outcomes by detecting early-stage cancers as pulmonary nodules. As most are benign, diagnosing these nodules is challenging and often requires surveillance imaging. The aim of this study is to assess the frequency of cancer progression in a lung cancer screening study as measured by tumour stage (T-stage).

**Methods:**

SUMMIT is a prospective cohort study assessing implementation of lung cancer screening with LDCT in a high-risk population. Screen-detected lung cancers with clinical tumour stage cT1a–c at time of referral were included. Upstaging was defined as an increase in T-stage from referral to treatment. The date of death was obtained from the National Cancer Registration and Analysis Service. Cox proportional hazards analysis with adjustment for age, sex, Charlson Comorbidity Index and pack years was used to assess mortality between groups.

**Results:**

390 screen-detected cancers were stage cT1a–c at time of referral. Upstaging occurred more frequently in cT1a (n=48, 56%) compared with cT1b (n=83, 38%) or cT1c (n=34, 40%), p=0.01. The proportion of part-solid nodules was similar between groups (upstaged-N=47, 27%, vs not upstaged-N=45, 21%, p=0.19). 43% of tumours increased T-stage from referral to first treatment (n=165). In participants upstaged, time from referral to treatment was longer (upstaged: 84 days, 46–298 vs not upstaged: 72 days, 43–211, p=0.04). Adjusted overall survival analyses showed an association between upstaging and mortality (HR 1.68, 95% CI 1.13 to 2.51, p=0.01).

**Conclusion:**

Tumour upstaging occurred in nearly half of early-stage cases in a lung cancer screening population. Tumour upstaging was associated with longer time to treatment and poorer outcomes in this population.

**Trial registration number:**

NCT03934866.

**WHAT IS ALREADY KNOWN ON THIS TOPIC**

- Low-dose CT screening enables early detection of lung cancer in high-risk patients which improves survival.

**WHAT THIS STUDY ADDS**

- A significant proportion of early-stage screen-detected lung cancers have an increase in tumour stage from referral to treatment (43%). This cancer upstaging is associated with longer care pathways and increased mortality.

**HOW THIS STUDY MIGHT AFFECT RESEARCH, PRACTICE OR POLICY**

- Further research into causes of delays within screening pathways and prognostication of detected pulmonary nodules is needed to optimise the efficiency and outcomes of lung cancer screening programmes.

## Introduction

 Lung cancer is the biggest cause of cancer-related mortality worldwide.^1^ This has been attributed to its frequent presentation at late stage.^2^ Over the last decade, there has been a focus on researching and implementing low-dose CT scan (LDCT) to screen for lung cancer in high-risk patients.^3–5^ Lung cancer screening has increasing evidence to support its implementation across health systems, with several countries now offering lung cancer screening programmes or recommending population screening.^6^

A key outcome from lung cancer screening studies is the concept of ‘stage-shift’ seen in screen-detected cancers compared with those clinically detected. Prior to lung cancer screening, only 28% of lung cancers in the UK were diagnosed at an early stage (stage I–II),^2^ yet in screening trials, nearly 70% are diagnosed at stage I.^3–5 7^

The reduction in lung cancer mortality is driven by the detection of early-stage cancers, for which therapies with curative intent can be given, including surgical resection or radiation therapy. Survival among individuals with stage IA ranges from 92% in individuals with a tumour <10 mm (IA1) to 77% in individuals with tumours sized 20–30 mm (IA3).^8^ This difference between stage IA1 and IA3 is related to only tumour size, suggesting that larger tumours are associated with poorer outcomes. Further, there is evidence that diagnostic delay in lung cancer is associated with a decline in patient survival.^9^ This is supported by evidence in early-stage lung cancers detected outside of screening suggesting that delays in diagnosis, inherent in pulmonary nodule guidelines designed to manage the high frequency of non-malignant nodules, can lead to growth of cancers and impact survival.^10^

Sequential evaluation of pulmonary nodules is an essential feature of an LDCT screening programme, though no previous studies have specifically reported on potential changes in cancer stage during the process. LDCT scanning has very high sensitivity with appropriate resolution to identify very small nodules.^11 12^ However, nodule detection is not specific to lung cancer, with many patients having fluctuating inflammatory nodules or nodules from other benign causes.^12^ Often with small nodules, follow-up imaging is required to detect growth, prior to a diagnostic work-up or treatment.^13^ This approach helps ensure a balance between early identification of lung cancer and minimisation of unnecessary intervention in benign nodules. Nevertheless, such an approach carries the risk that small lung cancers will grow during surveillance or diagnostic work-up. Although overall lung cancer screening does lead to a downwards stage shift compared with no screening at all, it is possible that screening is not yet diagnosing lung cancer at its earliest possible stage.

The aim of this study is to assess the frequency of tumour stage change during evaluation of screen-detected lung cancers and to assess factors associated with tumour upstaging. The results from this study can help to optimise lung cancer pathways and identify gaps in current research, to ensure the benefits of lung cancer screening are fully realised.

## Methods

The SUMMIT study is a prospective cohort study assessing the implementation of lung cancer screening within a high-risk population and validating a multicancer early detection blood test. Full details of the SUMMIT study have previously been published.^5 14 15^

### Cohort definition

Individuals diagnosed with screen-detected lung cancer, either at baseline LDCT or following surveillance, were identified in the SUMMIT population. LDCT scans at the time of referral for a suspicious pulmonary nodule were reviewed and clinical tumour stage (cT-stage) assigned based on characteristics recorded in the radiology reports. Individuals with screen-detected lung cancer initially referred with cT-stage T1a–c were included. The final cancer stage was determined at the time of treatment initiation. For surgical cases, the final T-stage was defined as the pathologic T-stage from the surgical specimen. For participants not undergoing surgery, clinical T-stage at the time of treatment decision was used, obtained from lung cancer multidisciplinary team medical record. In this analysis, ‘upstaging’ was defined as an increase from referral T-stage to final T-stage at treatment initiation. A confirmed lung cancer diagnosis was defined as the first pathology result demonstrating lung cancer. If cytopathologic confirmation was not available, then the treatment decision date was used as date of diagnosis. This definition was defined in the SUMMIT protocol but differs from the European National cancer registries, which use the positron emission tomography (PET) date or the lung multidisciplinary team (MDT) date as date of diagnosis. Given the often longer follow-up of pulmonary nodules, treatment decision date was chosen in SUMMIT as a surrogate to mark when the MDT definitively diagnosed cancer.

### Data collection

After eligibility assessment,^14^ participants underwent a clinical assessment and were consented to enrol in the SUMMIT study. Baseline participant characteristics including age, sex, height, weight, ethnicity, smoking status and total smoking pack years were collected at this visit. Index of multiple deprivation (IMD), based on postal code at consent, was also recorded and grouped into quintiles (1—most socioeconomic deprivation and 5—least deprived). PLCO_m2012_ (prostate, lung, colorectal, ovarian trial model)^16^ was calculated to predict 6-year risk of lung cancer incidence. Individuals were considered eligible if meeting United States Preventative Task Force 2013 criteria^17^ or PLCO_m2012_ ≥1.3%. Participants then underwent LDCT scan and nodule management followed modified BTS guidelines,^13^ with details on this nodule management protocol previously published.^18^ In brief, at baseline, solid nodules with a volume of ≥300 mm^3^ (or ≥8 mm diameter when reliable segmentation could not be achieved) and a Brock score of ≥10% were referred to a lung cancer multidisciplinary team for definitive investigation. Solid nodules with volumes of 80–300 mm^3^ (or ≥300 mm^3^ with Brock score <10%), consolidation, all part-solid and endobronchial nodules were considered indeterminate and underwent 3-month interval nodule follow-up CT. All pure ground glass nodules underwent interval surveillance at Y1. At the 3-month nodule follow-up CT, growing solid nodules with volume ≥200 mm^3^, persisting consolidation, endobronchial nodules and part-solid nodules with a solid component of ≥8 mm, increasing solid component or altered morphology were referred to the multidisciplinary team for further assessment. Participants with stable nodules at nodule follow-up CT underwent further scans at Y1 and Y2. Pulmonary nodules could remain under 3-month or 6-month surveillance within the SUMMIT study but were referred onward to multidisciplinary teams when lung cancer work-up was indicated. Lung cancer work-up was completed at 14 participating hospitals in the North-East and Central London area. Diagnostic decisions regarding further imaging evaluation, biopsy and treatment were at the discretion of the local clinical team. Data collected from these sites, including clinical TNM (tumour, nodal and metastasis) stage prior to treatment, pathological TNM stage, nodule density, interval imaging (including PET-CT and CT chest), biopsy details and treatment details.

Mortality data, underlying cause of death and co-morbidities were collected from National Cancer Registration and Analysis Service (NCRAS), NHS England in December 2024. This database provided date of death for participants with lung cancer and date at time of data extraction used for censoring. Comorbidity data provided by NCRAS included the total Charlson Comorbidity Index (CCI),^19^ calculated using hospital encounter data 6–78 months preceding diagnosis.^20^

### Statistical analysis

Descriptive statistics are reported as number, n, (percent %), mean±SD and median (10th–90th percentiles). Comparisons between participants with and without upstaging were completed with t-tests and analysis of variance (ANOVA) for parametric continuous data; Kruskal-Wallis testing for non-parametric continuous data; and χ^2^ testing for categorical data. Statistical significance was defined as a two-sided test with p<0.05 and 95% CIs are presented. Missing data were determined to be missing completely at random based on descriptive analyses of missingness patterns. Given data were missing completely at random and <5% of data were missing, listwise deletion was used in analyses, when applicable.

Logistic regression was used to assess the impact of participant factors (age, sex, cigarette pack years, CCI, IMD and ethnicity) and nodule characteristics (cT-stage at referral, density, time from referral to treatment) on upstaging. The log of time was used for analysis, given the right-skewed distribution of diagnostic time in this cohort.

To assess the impact of upstaging on lung cancer-specific and overall survival, competing risk and Cox proportional hazard models were used with adjustment for potential confounding factors including age, sex, cigarette pack years, CCI, IMD score and ethnicity (grouped as white and non-white for analysis). These confounders were selected due to known association with the outcome. Two sensitivity analyses were completed: (1) adjusted competing risk and cox proportional hazard models including only participants without nodal or distant metastasis at time of diagnosis (N0M0) and (2) adjusted cox proportional hazard models with the addition of log diagnostic time. Unadjusted subgroup survival analyses based on cT-stage at referral (cT1a, cT1b and cT1c) and final T-stage (T1b and T1c) were performed using log-rank tests and illustrated with Kaplan-Meier curves.

## Results

### Baseline characteristics

**Figure 1:**
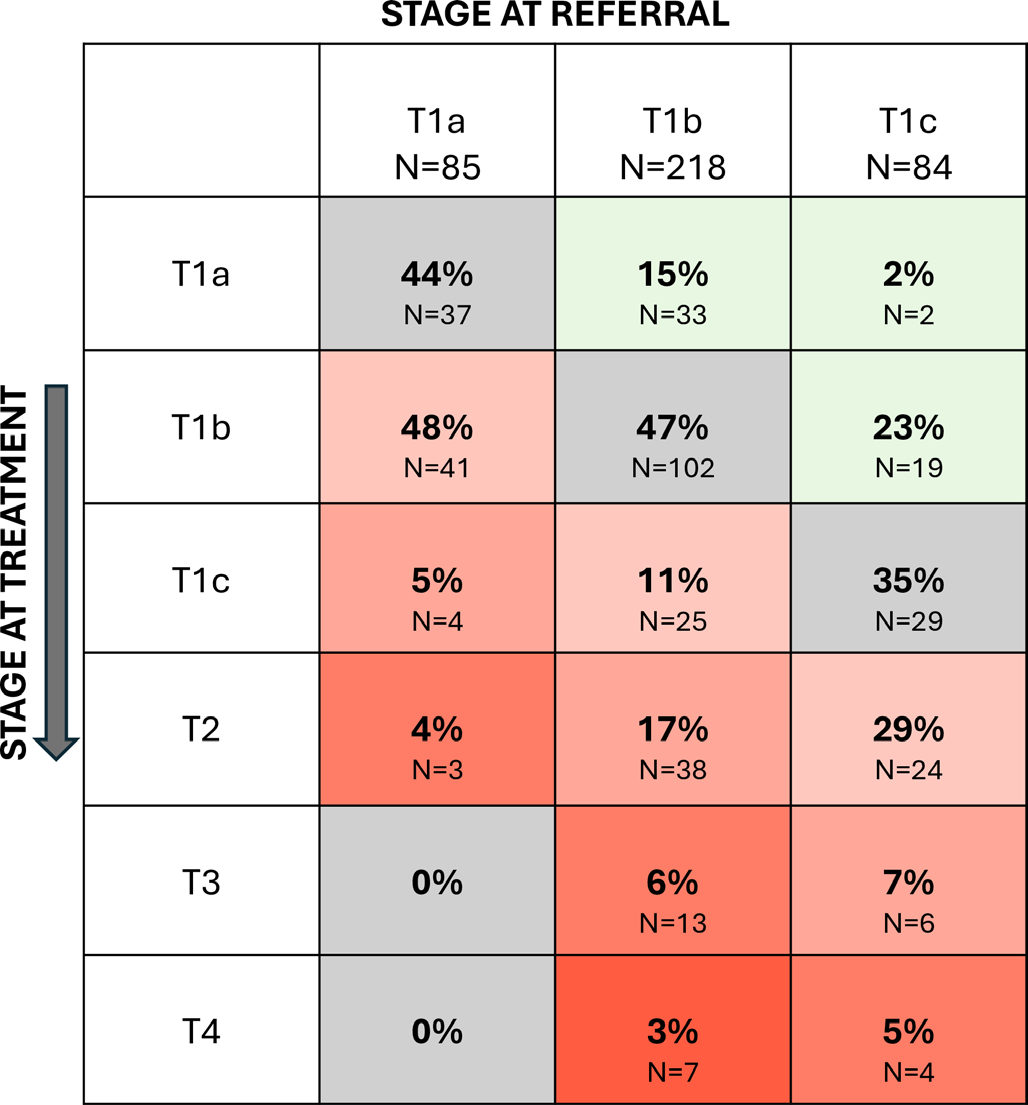
Comparison of tumour stage at referral and time of first treatment in screen-detected early-stage lung cancers. This figure demonstrates the frequency and proportion of upstaged tumours in screen-detected lung cancers separated by clinical tumour stage at time of referral. Red shading represents increased tumour stage (T-stage) from referral to treatment (final T-stage), whereas grey represents no change. Green shading indicates that clinical T-stage at referral over-estimated final T-stage. The majority of upstaging occurs by changing one to two t-stage categories. N=3 participants are excluded as final T-stage is unknown.

Up to December 2024, 390 participants were diagnosed with lung cancer after referral for pulmonary nodules clinical T-stage cT1a–cT1c: (cT1a: n=87 (22%), cT1b: n=219 (56%), cT1c: n=84 (22%)). The majority of nodules were solid (n=278, 71%) with fewer participants referred due to part-solid nodules (n=93, 24%). There was a tumour upstage between referral and treatment in n=165 (43%) of participants and n=54 (14%) had a tumour downstage, figure 1. Upstaged tumours occurred more frequently in those referred at cT1a (n=48, 56%) compared with cT1b (n=83, 38%) or cT1c (n=34, 40%), p=0.01.

Final tumour stage was based on pathologic staging in n=316 (82%) of participants, n=137 (83%) of those who upstaged and n=179 (81%) of those who did not, p=0.55. There was no difference in baseline participant or nodule characteristics between those who did and did not have tumour upstaging, table 1. In participants who upstaged, the proportion of part-solid nodules was similar to those who did not upstage (upstaged-N=45, 27%, vs not upstaged-N=47, 21%, p=0.19).

**Table 1:** Baseline characteristics in participants with and without tumour upstaging during assessment for screen-detected lung cancer

Characteristics	All participants, n=390	Upstaged, n=165*	Not upstaged, n=222*	P value
Age	68±6	67±6	68±6	0.23
Male	195 (50%)	83 (50%)	109 (49%)	0.82
CCI score total				0.79
0	215 (55)	95 (58)	118 (53)	
1	74 (19)	29 (18)	45 (20)	
2	32 (8)	12 (7)	20 (9)	
3	29 (7)	14 (8)	15 (7)	
≥4	40 (10)	15 (9)	24 (11)	
Cigarette pack years	45 (26–75)	46 (26–83)	44 (26–70)	0.46
PLCO_m2012_ percent	6.8±5.5	6.4±5.1	7.1±5.7	0.26
IMD quintile				0.16
1	101 (26)	49 (30)	51 (23)	
2	121 (31)	56 (34)	64 (29)	
3	86 (22)	28 (17)	58 (26)	
4	63 (16)	24 (15)	38 (17)	
5	17 (4)	8 (5)	9 (4)	
Missing	2 (1)	0 (0)	2 (0)	
Ethnicity				0.32
Asian	22 (6)	11 (7)	11 (5)	
Black	7 (2)	2 (1)	5 (2)	
White	349 (90)	144 (87)	202 (91)	
Multiple ethnicities	7 (2)	4 (2)	3 (1)	
Other	5 (1)	4 (2)	1 (0)	
Nodule density				0.19
Solid	278 (71)	115 (70)	161 (73)	
Part-solid	93 (24)	45 (27)	47 (21)	
Other†	19 (5)	5 (3)	14 (6)	

Data are presented as mean±SD, frequency (%) and median (10th–90th percentile).

* N=3 participants are excluded from upstaged and not upstaged categories, as final T stage was missing.

† Nodule density group other includes pure ground glass nodules and calcified nodules. There was no difference in upstaging based on hospital trust of first evaluation (data in online supplemental table S3).

CCI, Charlston Comorbidity Index; IMD, index of multiple deprivation; N, number; PLCO_m2012_, prostate, lung, colorectal and ovarian model.

### Diagnostic pathway outcomes

Median time from referral LDCT to diagnosis was 60 days (21–216) for all participants. This was longest for participants with cT1a at referral (cT1a: 71 days (29–298), cT1b: 60 days (21–209) and cT1c: 47 days (16–138), p<0.01). Time from referral LDCT to diagnosis was higher in participants who had tumour upstaged (67 days, 22–385) compared with those who had not upstaged (56 days, 20–183) though this did not meet statistical significance (p=0.11).

There was no difference in the frequency of in-study LDCT for nodule surveillance (3 or 6 months) prior to lung cancer referral between groups (upstaged n=49, 30% vs not upstaged n=75, 34%, p=0.39). There was additional surveillance imaging after referral in n=53 (32%) of participants who upstaged and n=57 (26%) of participants who did not upstage, though this did not meet statistical significance, p=0.16.

There was no difference in the rate of non-surgical biopsy attempt prior to diagnosis between groups (upstaged-N=95, 58% vs not upstaged-N=114, 51%, p=0.22). There was no difference in the proportion of patients with diagnostic non-surgical biopsy between groups (upstaged-47% n=77 vs not upstaged-45% n=99, p=0.69). Time to diagnostic biopsy was slightly longer in the group that upstaged, though this did not meet statistical significance (upstaged 43 days, 17–187 vs no upstaging 37 days, 14–159, p=0.34).

More participants with tumours that upstaged had nodal involvement at time of treatment (upstaged-N=42, 25% vs not upstaged-N=21, 10%, p<0.01). Metastatic disease was also more common in participants with tumours that upstaged at time of treatment (upstaging-N=13, 8% vs no upstaging-N=5, 2%, p=0.02). Nodal involvement, metastatic disease and overall final staging are shown in table 2. As well, n=51 (31%) participants who upstaged had evidence of pleural invasion on pathology.

**Table 2:** Final TNM 8th edition staging of screen-detected lung cancers in participants with and without tumour upstaging

Final stage	All lung cancers detected by screening, n=390	Upstaged, n=165*	Not upstaged, n=222*	P value
Final T-stage				.
T1a	72 (18)	.	72 (32)	
T1b	162 (42)	41 (25)	121 (55)	
T1c	58 (15)	29 (18)	29 (13)	
T2	65 (17)	65 (40)	.	
T3	19 (5)	19 (12)	.	
T4	11 (3)	11 (7)	.	
Unknown	3 (1)	.	.	
Final N-stage				<0.01
N0	325 (84)	123 (76)	201 (91)	
N1	20 (5)	14 (9)	6 (3)	
N2	31 (8)	20 (12)	11 (5)	
N3	13 (3)	8 (5)	4 (2)	
Unknown	1 (0)	.	.	
Final M-stage				0.02
M0	371 (95)	152 (92)	217 (98)	
M1a	10 (3)	9 (6)	1 (0)	
M1b	6 (2)	3 (2)	2 (1)	
M1c	3 (1)	1 (0)	2 (1)	
Overall stage				<0.01
I	310 (80)	112 (68)	197 (89)	
II	21 (5)	16 (10)	5 (2)	
III	40 (10)	25 (15)	15 (7)	
IV	18 (5)	12 (7)	5 (2)	
Unknown	1 (0)	0 (0)	0 (0)	

Data are presented as frequency (%).

* N=3 participants are excluded from upstaged and not upstaged categories, as final T stage was missing.

M, metastasis; N, nodal; n, number; T, tumour.

### Treatment outcomes

There was no difference in the rate of surgery between groups. Fewer participants who upstaged received curative intent radiotherapy, with more participants receiving combined chemoradiotherapy or palliative chemotherapy (p<0.01, table 3).

**Table 3:** Types of treatment for screen-detected lung cancers in participants with and without tumour upstaging

First treatment	All lung cancers detected by screening, n=390	Upstaged, n=165	Not upstaged, n=222	P value
Neoadjuvant	3 (1)	1 (0.6)	2 (1)	<0.01*
Curative surgery	316 (81)	134 (81)	182 (82)	
Curative radiotherapy	36 (9)	9 (5)	25 (11)	
Chemoradiotherapy	10 (3)	6 (4)	4 (2)	
Palliative radiotherapy	1 (0.3)	0	1 (0)	
Palliative systemic	16 (4)	13 (8)	2 (1)	
None	7 (2)	2 (1)	5 (2)	
Unknown	1 (0)	0	1 (0)	

Data is presented as frequency (%).

* P value represents grouping of palliative therapies and exclusion of none/unknown.

n, number.

**Figure 2:**
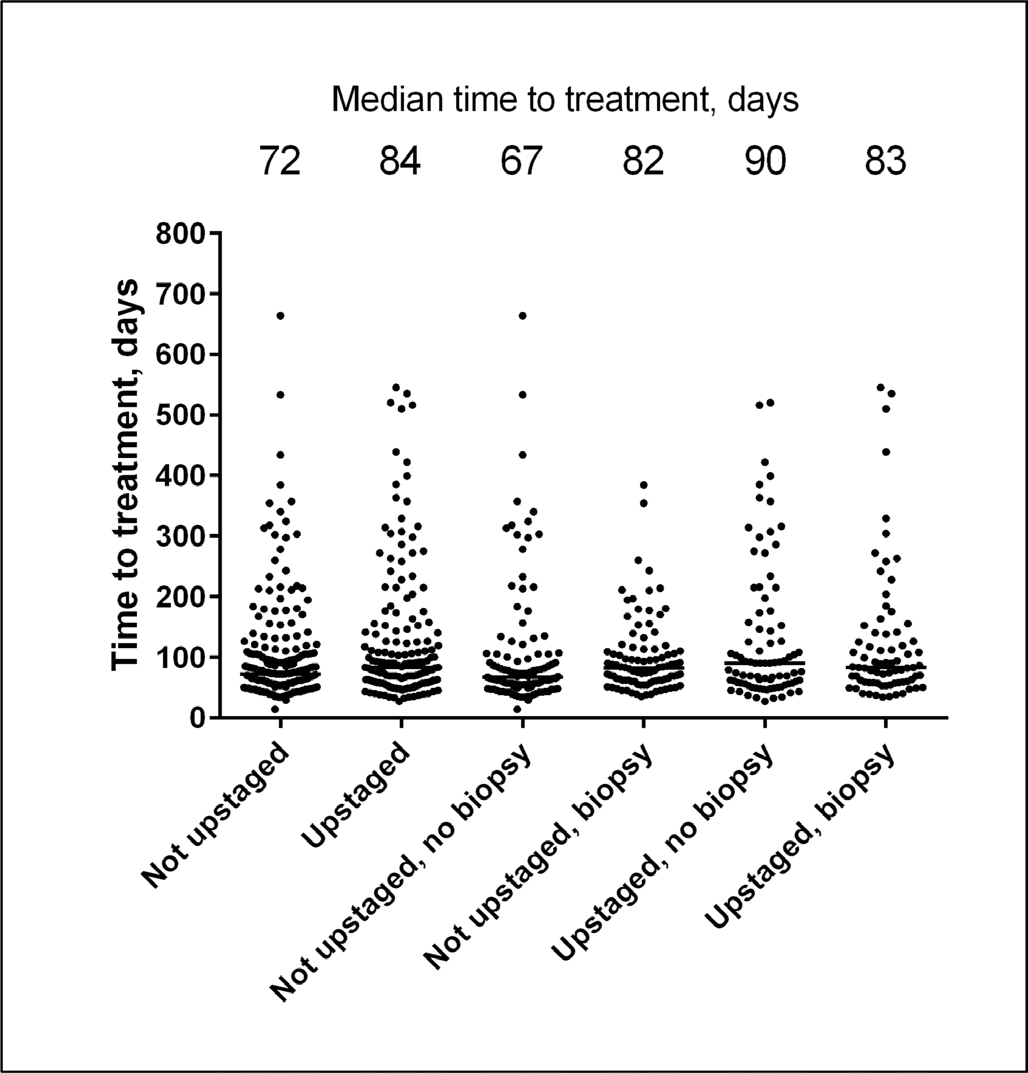
Median time from referral to treatment stratified by upstaging and diagnostic biopsy in screening participants with malignant pulmonary nodules. This figure demonstrates the median time between referral from the screening programme to a clinical team for lung cancer work-up and final lung cancer diagnosis in those with pulmonary nodules ≤30 mm (T1) in the SUMMIT study. It shows longer and more variable time to diagnosis in those with tumours that upstaged.

Overall, time from referral to treatment was longer in participants who upstaged compared with those who did not (upstaged 84 days, 46–298 vs no upstaging 72 days, 43–211, p=0.04), figure 2. For participants with a definitive diagnosis prior to surgery (n=176, 45%), there was no difference in time from diagnosis to treatment between participants who did and did not upstage (upstaged 83 days, 47–263 vs not upstaged 82 days, 47–180 days, p=0.54). In participants without a definitive diagnosis prior to treatment (n=211, 55%), there was a longer treatment delay in those that upstaged (upstaged 90 days, 45–314 vs no upstaging 67 days, 42–278, p=0.04). In multivariate regression analysis, time to treatment was associated with upstaging (OR 1.56 95% CI 1.09 to 2.21, p=0.01), while higher referral T-stage was less associated with upstaging, though this did not meet statistical significance, table 4.

**Table 4:** Logistic regression assessing the association of participant and nodule characteristics with tumour upstaging

Variable	OR	95% CI	P value
Age	0.98	0.94 to 1.02	0.27
Male	0.93	0.59 to 1.46	0.74
Cigarette pack years	1.01	1.00 to 1.02	0.08
CCI score	0.96	0.81 to 1.15	0.67
IMD score	1.00	1.00 to 1.00	0.20
Ethnicity			0.47
White	Reference		
Other ethnicity	1.30	0.63 to 2.68	
Nodule density			0.76
Solid	Reference		
Subsolid	1.09	0.65 to 1.83	
Referral cT-stage			
1a	Reference		
1b	0.58	0.33 to 1.03	0.06
1c	0.73	0.37 to 1.47	0.38
Time to treatment	1.56	1.09 to 2.21	0.01

Time to treatment is included as log of time to treatment in this analysis given its skewed distribution.

CCI, Charlston Comorbidity Index; cT-stage, clinical tumour stage; IMD, index of multiple deprivation.

### Survival

**Figure 3:**
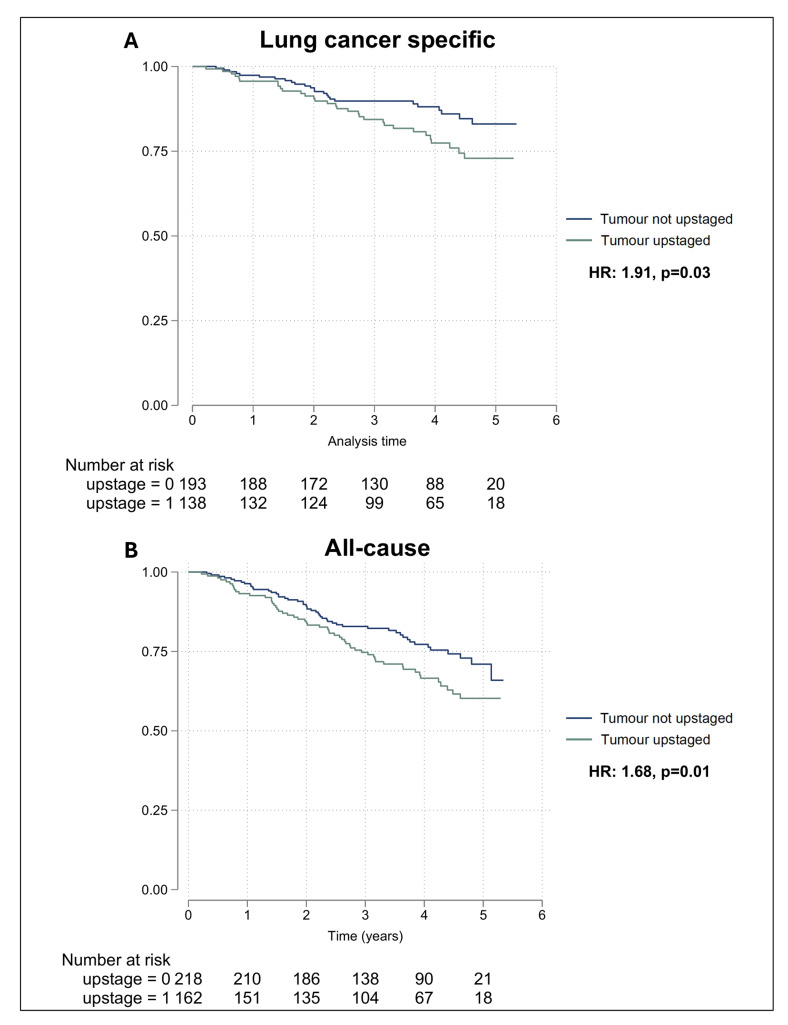
Survival for participants diagnosed with lung cancer with and without tumour upstaged after referral. This figure demonstrates (**A**) lung cancer-specific and (**B**) all-cause mortality from time of referral in participants in the SUMMIT lung cancer screening study with and without tumour upstaging. HRs are adjusted for age, sex, cigarette pack years, CCI, IMD score and ethnicity (grouped as white and non-white for analysis). Median follow-up time was 3.7 years from referral. CCI, Charlson Comorbidity Index; IMD, index of multiple deprivation.

n=107 participants (27%) died from any cause during follow-up with n=57 (53%) of deaths attributed to lung cancer (cause of death is shown in online supplemental table S1). Median follow-up was 3.7 years from lung cancer referral and 4.8 years from the date of SUMMIT study consent. When adjusting for T-stage at time of referral, age, sex, pack years, CCI, IMD and ethnicity, there was an association between tumour upstage from referral to treatment and lung cancer specific mortality and all-cause mortality (lung cancer specific-HR 1.91, 95% CI 1.09 to 3.35, p=0.03 and all-cause HR 1.68, 95% CI 1.13 to 2.51, p=0.01), table 5. Overall and lung cancer-specific mortality is shown in Kaplan-Meier graphs for all participants compared by upstaging, figure 3.

**Table 5:** Adjusted competing risk and cox-proportional hazard models demonstrating mortality risk in participants with tumour upstaging during lung cancer work-up

Variable	Lung cancer specific			All-cause		
	HR	95% CI	P value	HR	95% CI	P value
Upstaged	1.91	1.09 to 3.35	0.03	1.68	1.13 to 2.51	0.01
Referral cT-stage						
1a	Reference			Reference		
1b	6.30	1.49 to 26.53	0.01	1.94	1.03 to 3.65	0.04
1c	11.86	2.78 to 50.86	<0.01	3.35	1.73 to 6.50	<0.01
Age	1.03	0.98 to 1.08	0.32	1.03	0.99 to 1.07	0.14
Male	1.22	0.69 to 2.17	0.50	1.56	1.03 to 2.36	0.04
CCI score	1.15	0.98 to 1.35	0.08	1.24	1.11 to 1.39	<0.01
Cigarette pack years	1.00	0.99 to 1.01	0.46	1.00	0.99 to 1.00	0.64
IMD score	1.00	1.00 to 1.00	0.48	1.00	1.00 to 1.00	0.51
Ethnicity						
White	Reference			Reference		
Other ethnicity	0.55	0.17 to 1.83	0.33	0.72	0.35 to 1.45	0.36

CCI, Charlston Comorbidity Index ; cT-stage, clinical tumour stage; IMD, index of multiple deprivation.

In adjusted sensitivity analyses including only participants with no pleural invasion, nodal or metastatic disease at time of diagnosis (N0M0), tumour upstaging was still associated with higher lung cancer specific and all-cause mortality, though lung cancer specific mortality did not reach statistical significance (lung cancer specific HR 1.79, 95% CI 0.99 to 3.23, p=0.052 and all-cause HR 1.67, 95% CI 1.10 to 2.53, p=0.02), online supplemental figure S1 and table S2. Upstaging is likely to influence the choice of treatment, and together with other clinical decisions that may not have been captured as variables, would affect survival. Therefore, treatment was not included in the main regression models. However, if we add treatment to the multivariable regression, the adjusted HR for upstaging is still high (lung cancer specific HR 1.59, 95% CI 0.89 to 2.85 and all-cause HR 1.62, 95% CI 0.91 to 2.89).

Kaplan-Meier graphs with log-rank tests for subgroup analysis of each cT-stage at referral (online supplemental figure S2) and each final T-stage (online supplemental figure S3) according to whether or not they were upstaged are shown in the online supplemental appendix. These figures suggest differences in mortality may be driven by upstaging of cancers referred at T1b and T1c, though event rates in cancers referred at T1a are limited. When grouping by final T-stage, upstaged tumours have similar outcomes to tumours that did not upstage.

## Discussion

This study highlights that tumour growth and T-stage increase is common during assessment of screen-detected lung cancers. Nearly half of screen-detected cancers had upstaging prior to treatment. Upstaging was associated with small size, as it was more common in nodules referred at cT1a, but did not have an association with nodule density, as similar proportions of solid and part-solid nodules upstaged compared with those that did not. It is possible that upstaging in T1a nodules was more common due to greater diagnostic uncertainty and difficulty in obtaining a definitive diagnosis at a size <10 mm. These lesions are often monitored clinically for growth, which correlates with higher frequency of upstaging. The proportion of upstaging within a screening cohort has not previously been reported and may be an important metric to help optimise future programmes and research.

This study demonstrates delays from referral to treatment in screen-detected lung cancer, with the longest delay in those referred for pulmonary nodules<10 mm. These longer delays were associated with T-stage increase in this study, when adjusting for confounders. Although the exact cause of delays could not be determined, factors such as longer time to diagnosis, biopsy, treatment (especially in those without a diagnostic biopsy), and more frequent follow-up imaging in upstaged patients may suggest challenges in confirming the diagnosis. Although we show a clear association between time to treatment and upstaging, we cannot conclude a causal link as there could be unmeasured confounding relating to the participants’ health status or other factors that may influence appropriate timely diagnostic work-up and survival. It is also possible that system level factors such as resource limitations of biopsy, imaging and specialist clinics could have contributed to delays. However, given the factors we examined, we can say that delays were independently associated with upstaging, which would suggest that optimising timeliness is essential.

This study is the first to demonstrate that a change in T-stage is associated with poorer outcomes, even within a screening population. Tumour upstaging was associated with increased mortality. Though the eventual diagnosis of pleural invasion, nodal or metastatic disease was more common in participants with tumours that upstaged, the association between tumour upstaging and increased mortality was still present in sensitivity analysis that excluded patients with pleural invasion or N/M>0. This suggests that tumour growth and T-stage change during evaluation is in itself associated with survival and an important prognostic factor for lung cancer outcomes.

With lung cancer screening, we will continue to identify small pulmonary nodules that will require diagnostic work-up. Previously, it has been shown that small, growing nodules carry higher risk of lung cancer,^21^ and it has also been shown that delays due to COVID-19 within screening programmes were associated with diagnosis at advanced stage.^22^ Evidence from studies modelling growth suggests that small growing pulmonary nodules are associated with worse survival, and delays in this diagnosis again were associated with worse outcomes.^23 24^ Our findings suggest that even within screening programmes, delays impact patient outcomes and should be minimised.

Delays could be due to resource limitation and systematic barriers within cancer pathways but may also relate to diagnostic uncertainty. In this study, upstaging was most common in T1a nodules and in multivariate analysis, increasing T-stage was inversely related to upstaging. Current technology and tools may be insufficient to evaluate small pulmonary nodules and confirm these cases at the earliest possible stage. Expanding these tools is essential to balance the harms of delayed diagnosis against the harms of invasive diagnostic procedures for benign nodules. Further research into tools for early diagnosis of lung cancer is ongoing with advances in robotic-assisted bronchoscopy and ongoing research into AI-prediction models and biomarkers.^25–29^ Identifying high-risk features for small pulmonary nodules before demonstration of rapid growth is essential in ensuring diagnosis at the earliest stage, without exposing patients to invasive procedures for benign nodules.

This study has several strengths that add to the body of evidence in lung cancer screening. It is the first study to describe tumour upstaging within a screening programme and the first to explore the impact on patient outcomes in this context. This cohort is derived from a large screening study with a high prevalence in early-stage cancers, allowing for the assessment of this subgroup.

There remain limitations within this study, including that a portion of the study occurred throughout the COVID-19 pandemic. This may have contributed to some diagnostic delay, with the impact on NHS service, and quantifying the contribution of this delay is not feasible. Another potential limitation is the inability to determine true biological progression compared with inaccurate clinical staging. This is particularly relevant for part solid nodules which are difficult to stage clinically; however, this represents a minority of lung cancers in this study and previous studies demonstrated that clinical stage may overestimate compared with pathologic stage.^30 31^ Further to this, 31% of participants who upstaged had evidence of pleural invasion, which explains at least in part T-stage increase to T2 and beyond. It is not possible to know if this was present at referral and may contribute to misclassification of upstaging. As such, these participants were excluded in sensitivity analyses in an effort to isolate true biologic progression, though this cannot be definitively confirmed and remains a limitation.

Overall, the goal of lung cancer screening is to diagnose lung cancer at an earlier stage, leading to a stage shift and improved survival. Although this is still happening on a large scale compared with clinically diagnosed cancer, tumour upstaging in screening is occurring at a high rate. Upstaging has a meaningful impact on patient outcomes and may limit the cost-effectiveness of lung cancer screening. Further study into causes of delays in lung cancer screening programmes is pivotal to optimising patient outcomes and improving care. In addition, future studies are needed to predict tumour growth and evaluate its potential as a prognostic factor to guide patient management.

## Supplementary material

10.1136/thorax-2025-224006 online supplemental file 1

10.1136/thorax-2025-224006 online supplemental file 2

## Data Availability

Data are available upon reasonable request.
