# Supplementary material for: Upstaging of screen-detected lung cancers during diagnostic assessment
Source: Thorax. 2026 Feb 2;81(9):e224006. doi: 10.1136/thorax-2025-224006 (PMC13479619; doi:10.1136/thorax-2025-224006)
Supplement: Supplementary file 1 [file thorax-81-9-s001.docx]

**Supplemental Appendix:**

**Supplemental table S1:** Underlying cause of death for study participants

| Cause^*^ | Frequency N(%) |
| --- | --- |
| Lung Cancer | 57 (53) |
| Other Cancer^1^ | 9 (8) |
| Cardiovascular disease^2^ | 9 (8) |
| Stroke or Peripheral vascular disease^3^ | 5 (5) |
| Respiratory disease^4^ | 18 (17) |
| Other/Unknown^5^ | 9 (8) |

Abbreviation: N, number.

*^*^Underlying cause of death is based on ICD-10 coding within NCRAS. Coding has been grouped, as some codes have N<5. ^1^Other cancer includes: Head and neck, pancreatic, breast, prostate, brain and unspecified; ^2^Cardiovascular disease includes myocardial infarction, coronary artery disease, cardiomyopathy, congestive heart failure and myocarditis; ^3^Stroke and peripheral vascular disease includes ischemic/haemorrhagic stroke, aortic dissection, and peripheral vascular disease, unspecified; ^4^Respiratory diseases include: emphysema, chronic obstructive pulmonary disease, acute exacerbation of chronic obstructive pulmonary disease; interstitial lung disease and coronavirus disease 2019; ^5^Other includes bowel perforation, alcoholic liver failure, and Guillain-Barré syndrome*

**Supplemental table S2:** Sensitivity analysis for lung cancer specific mortality in participants without nodal or distant metastasis at time of diagnosis

| **Variable** | **Lung cancer specific** | | | **All-cause** | | |
| --- | --- | --- | --- | --- | --- | --- |
|  | **Hazard ratio** | **95%CI** | **p-value** | **Hazard ratio** | **95%CI** | **p-value** |
| Upstaged | 1.75 | 0.97-3.17 | 0.065 | 1.67 | 1.10-2.53 | 0.02 |
| Referral cT-stage  1a  1b  1c | Reference  5.92  10.45 | 1.39-25.2  2.40-45.4 | 0.02  <0.01 | Reference  1.80  3.01 | 0.95-3.41  1.53-5.89 | 0.07  <0.01 |
| Age | 1.03 | 0.97-1.09 | 0.34 | 1.03 | 0.99-1.07 | 0.17 |
| Male | 1.36 | 0.74-2.50 | 0.32 | 1.57 | 1.02-2.42 | 0.04 |
| CCI score | 1.18 | 1.01-1.38 | 0.04 | 1.28 | 1.15-1.43 | <0.01 |
| Cigarette pack years | 1.00 | 0.99-1.01 | 0.67 | 1.00 | 0.99-1.01 | 0.59 |
| IMD score | 1.00 | 1.00-1.00 | 0.56 | 1.00 | 1.00-1.00 | 0.73 |
| Ethnicity  White  Other ethnicity | *Reference*  0.50 | 0.13-1.93 | 0.31 | *Reference*  0.63 | 0.28-1.40 | 0.25 |

Abbreviations: CI, confidence interval; cT-stage, clinical tumour stage; CCI, Charlston co-morbidity index ; IMD, index of multiple deprivation.

**Supplemental table S3:** Association of upstaging and first hospital trust

| Characteristics | Upstaged  N=165^*^ | Not upstaged N=222^*^ | p-value |
| --- | --- | --- | --- |
| Hospital Trust  A  B  C  D  E  F  G  H | 36 (41)  36 (44)  3 (60)  6 (32)  10 (45)  32 (37)  28 (54)  6 (35) | 57 (59)  45 (56)  2 (40)  13 (68)  12 (55)  55 (63)  24 (46)  11 (65) | 0.53 |

*Data presented as N(%) with trusts listed as A-H. N=20 participants had missing first trust data.*

**Supplemental Figure S1:** Sensitivity analysis of survival for participants without pleural invasion, nodal or distal metastasis with upstaged and without upstaged tumours

*This figure demonstrates lung cancer specific and all-cause mortality from time of referral in participants diagnosed with lung cancer that did not have pleural invasion, nodal or distal metastasis. Data is shown by those with upstaged and without upstaged tumours from stage at time of referral. Hazard ratios are adjusted for* age, sex, cigarette pack years, CCI, IMD score and ethnicity (grouped as white and non-white for analysis).  *Median follow up time was 3.7 years from referral.*

**Supplemental Figure S2:** Survival for participants diagnosed with lung cancer with and without tumour upstaging by tumour stage at time of referral

*This figure demonstrates lung cancer specific and all-cause mortality from time of referral in participants in the SUMMIT lung cancer screening study with upstaged and without upstaged tumours, by tumour stage at time of referral. Participants with T1a nodules at referral are represented in panels A-B, T1b in C-D and T1c in E-F. Median follow up time was 3.7 years from referral.*

**Supplemental Figure S3:** Survival for participants diagnosed with lung cancer with and without tumour upstaging by final tumour stage

*This figure demonstrates lung cancer specific and all-cause mortality from time of referral in participants in the SUMMIT lung cancer screening study with upstaged and without upstaged tumours, by final tumour stage. Participants with final T-stage of T1b are represented in panels A-B, and final T-stage T1c in C-D. Median follow up time was 3.7 years from referral.*

**The SUMMIT Consortium**

Aashna Samson⁴, Adefiola Olabode⁴, Alberto Villanueva⁴, Ali Mohammed¹³, Alice Cotton⁴, Andrew Crossingham⁴, Andrew Perugia²², Anjeli Ketkar⁴, Anna Sikorska⁴, Anthony Edey⁴, Antonette Andrews⁴, April Neville⁴, Burcu Ozaltin²⁰, Camilla Mitchell⁴, Catherine Nestor⁴, Celia Snell⁴, Charlie Sayer⁴, Charlotte Cash⁷, Chimtom Nwaosu⁴, Christine Hosein⁴, Claire Levermore⁴, Columbus Ife⁴, Derya Ovayolu⁴, Dominique Arancon⁴, Domminique Arancon⁴, Eleanor Hellier⁴, Elena Stefan⁴, Elodie Murali⁴, Esther Arthur-Darkwa⁴, Ethaar El-Emir⁴, Fabia Ruggiero⁴, Fahiza Chowdhury⁴, Fahmida Hoque⁴, Fanta Bojang⁴, Farhida Begum⁴, Geoff Bellingan⁴, Georgia Bullock⁴, Graham Robinson⁴, Gulistan Keskinbicak⁴, Gulten Geneci⁴, Harriet Sorrell⁴, Hasti Robbie⁴, Helen Kiconco⁴, Helen Victoria Bowyer⁴, Henna Makwana⁴, Hina Pervez⁴, Houda Benmebarek⁴, James Rusius²², Jane Rowlands⁴, Janine Zylstra⁴, Jeannie Eng⁴, Jhanara Begum⁴, Jon Teague², Joseph Jacob²⁰, Judy Airebamen⁴, Julian McKee⁴, Karen Parry-Billings⁴, Karen Sennett²³, Kate Davies¹, Kate Gowers¹, Kaylene Phua⁴, Kim Sorley⁴, Kitty Chan², Kylie Gyertson⁴, Laila Ahmed⁴, Laura Farrelly², Laura Green⁴, Lucas Clarke⁴, Lynsey Gallagher⁴, Magali Taylor⁴, Malavika Suresh², Mamta Ruparel¹, Marie Calligaris⁴, Marilyn Ijeomah-Orji⁴, Mark Clark⁴, Maureen Browne⁴, Mehran Azimbagirad²⁰, Miguel Ferreira⁴, Milica Rajkov⁴, Moksud Miah⁴, Monika Sommerrey⁴, Navinah Nundlall⁴, Nazim Miah⁴, Neil Songsong⁴, Nicholas Beech⁴, Nick Screaton¹⁹, Nikita Sharma⁴, Olufunmilola Ajayi⁴, Oluwadara Akerewusi⁴, Patricia Castello⁴, Paul Hickey⁴, Paul Robinson⁴, Qin Neville⁴, Rabiya Patel⁴, Rachael Sarpong², Ricardo McEwen⁴, Rosie Dapaah Tweneboah⁴, Rowan Faiers⁴, Sahana Uthayakumar⁴, Sam Hare⁴, Samanjit Hare⁷, Sara Lock⁸, Sebri Hasaballa⁴, Shaira Hassan⁴, Sheetal Karavadra⁴, Shrinkhala Dawadi², Shummi Begum⁴, Simranjit Mehta⁴, Sofia Nnorom⁴, Stephen Ellis⁴, Stuart Marnham⁴, Sumiya Mahey⁴, Suraiya Sharmin⁴, Syeda Anam Hussain⁴, Sylvia Patricia Enes⁴, Tania Anastasiadis²¹, Tanita Limani⁴, Tanya Patrick¹, Terry O'Shaughnessy¹³, Thea Buchan⁴, Theepan Visakan⁴, Thomas Callender¹, Tracy Odigie⁴, Trisha Enes⁴, Tunku Aziz¹³, Urja Patel⁴, Vicky Bowyer⁴, Ya-Fei Li⁴, Yee Chin Lee⁴, Zaheer Mangera¹²,Zahra Awan⁴, Zahra Hanif⁴, Zelena Aziz⁴, Robert Chapman²⁴, Daryl O. Cheng²⁴, Shahab Aslani²⁴, Adam Szmul²⁴, Yamada Daisuke²⁴, Ryoko Egashira²⁴, Shanshan Wang²⁴, Shiyu Wang²⁴, Pardeep Vasudev²⁴, Niccolo McConnell²⁴, Helen McGregor²⁴

^1^Lungs for Living Research Centre, UCL Respiratory, University College London, London

^2^CRUK & UCL Cancer Trials Centre, University College London, London

^4^University College London Hospitals NHS Foundation Trust, London

^7^Royal Free London NHS Foundation Trust, London

^8^ Whittington Health NHS Trust, London

^12^North Middlesex University Hospital NHS Trust, London

^13^Barts Health NHS Trust, London

^19^Royal Papworth Hospital NHS Foundation Trust, Cambridge

^20^Satsuma Lab, Centre for Medical Image Computing (CMIC), London

^21^Tower Hamlets Clinical Commissioning Group, London

^22^Noclor Research Support, London

^23^Islington Clinical Commissioning Group, London

^24^Satsuma Lab, UCL Hawkes Institute
